# Femtosecond laser semi-assisted Descemet stripping endothelial keratoplasty: 2-year outcomes of endothelial cell loss and graft survival

**DOI:** 10.1007/s00417-021-05383-x

**Published:** 2021-08-31

**Authors:** Ting Wang, Peiyan Shi, Fengjie Li, Hua Gao, Suxia Li, Tong Liu, Weiyun Shi

**Affiliations:** Department of Ophthalmology, Eye Hospital of Shandong First Medical University, State Key Laboratory Cultivation Base, Shandong Provincial Key Laboratory of Ophthalmology, Shandong Eye Institute, Shandong First Medical University & Shandong Academy of Medical Sciences, 372 Jingsi Road, Jinan, 250021 China

**Keywords:** Femtosecond laser, Descemet stripping endothelial keratoplasty, 2-year endothelial cell loss, Graft survival

## Abstract

**Purpose:**

To assess 2-year endothelial cell loss and graft survival after femtosecond laser semi-assisted Descemet stripping endothelial keratoplasty (FLS-DSEK).

**Methods:**

In this prospective and noncomparative study carried out at Eye Hospital of Shandong First Medical University, 85 eyes (84 patients) with endothelial dysfunction receiving FLS-DSEK (*n*=62, 75.9%) or FLS-DSEK combined with phacoemulsification cataract surgery and intraocular lens implantation (*n*=23, 27.1%) from 2013 through 2016 were included. The graft endothelial cell loss, endothelial graft thickness, visual acuity, and complications after surgery were evaluated.

**Results:**

Thin endothelial grafts were all successfully prepared, with no occurrence of perforation. The rate of endothelial cell loss was 17.4%, 18.8%, 19.9%, and 26.7%, and the central graft thickness was 113±54 μm, 102±40 μm, 101±28 μm, and 96±23 μm at 3, 6, 12, and 24 months, respectively. The median best-corrected visual acuity was 0.4 logMAR (range, 0–2 logMAR) at 24 months, demonstrating a significant difference from that before surgery (2 logMAR; range, 0.2–3 logMAR) (*T*=187.5, *P*<.001). Partial graft dislocation was the most common postoperative complication, with an occurrence rate of 14% (*n*=12), and it was associated with an abnormal iris-lens diaphragm (*r*=.35, *P*<.001). The other complications included a high intraocular pressure (*n*=5, 6%), endothelial graft rejection (*n*=4, 5%), and pupillary block (*n*=1, 1%). Endothelial graft decompensation occurred in the two eyes, and 98% (*n*=83) of the grafts survived at 24 months.

**Conclusions:**

Data of the study suggest that the treatment using FLS-DSEK seems to be promising and might be considered a feasible choice in patients with endothelial dysfunction.

**Trial registration:**

1. Date of registration: 2021-02-18

2. Trial registration number: ChiCTR2100044091

3. Registration site: https://www.chictr.org.cn/

**Supplementary Information:**

The online version contains supplementary material available at 10.1007/s00417-021-05383-x.

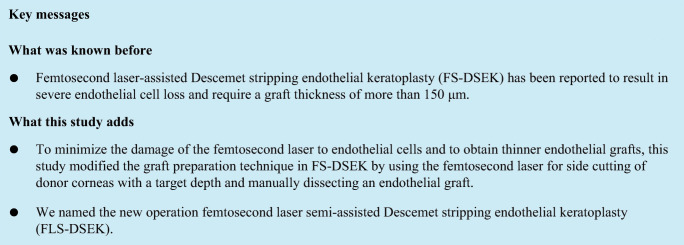



## Introduction

Endothelial keratoplasty has developed rapidly and becomes a major surgical approach to corneal endothelial dysfunction [1–3] since Descemet stripping endothelial keratoplasty (DSEK) was first introduced in 2005 [4]. Currently, Descemet membrane endothelial keratoplasty (DMEK) seems to be most suitable for the anatomical structure of the cornea. However, due to the more challenging preparation of donor tissue [5, 6], and the limited number of high quality donor corneas [7], this procedure has not been widely accepted in China [8]. Although the preparation of an endothelial graft using a microkeratome carries a high rate of success [9], the risk of perforation persists when an ultrathin endothelial graft is needed [10, 11]. Cheng et al. [12] employed a femtosecond laser for cutting an endothelial graft in 2007, making the preparation of the graft more convenient, unified, and controllable. Nevertheless, femtosecond laser-assisted Descemet stripping endothelial keratoplasty (FS-DSEK) has been reported to result in severe endothelial cell loss [13], thus requiring a graft thickness of more than 150 μm [14]. To minimize the damage of the femtosecond laser to endothelial cells and to obtain thinner endothelial grafts, we modified the graft preparation technique in FS-DSEK by using the femtosecond laser for side cutting of donor corneas with a target depth and manually dissecting an endothelial graft. We named the new operation femtosecond laser semi-assisted Descemet stripping endothelial keratoplasty (FLS-DSEK).

## Materials and methods

### Inclusion criteria

Eighty-four consecutive adult patients (85 eyes) with endothelial dysfunction receiving FLS-DSEK (62 eyes) or FLS-DSEK combined with phacoemulsification cataract surgery and intraocular lens (IOL) implantation (23 eyes) by the same surgeon (W.S.) at Eye Hospital of Shandong First Medical University from May 2013 through April 2016 were included in the analysis. In addition to endothelial dysfunction, the patients also suffered from other eye diseases. This prospective study was approved by the Institutional Review Board of Eye Hospital of Shandong First Medical University (No. 201302) and adhered to the tenets of the Helsinki Declaration. Written informed consent was obtained from each patient.

### Surgical technique (online resource)

In patients requiring combined surgery, phacoemulsification and IOL implantation were performed before FLS-DSEK.

### Endothelial graft preparation

**Fig. 1:**
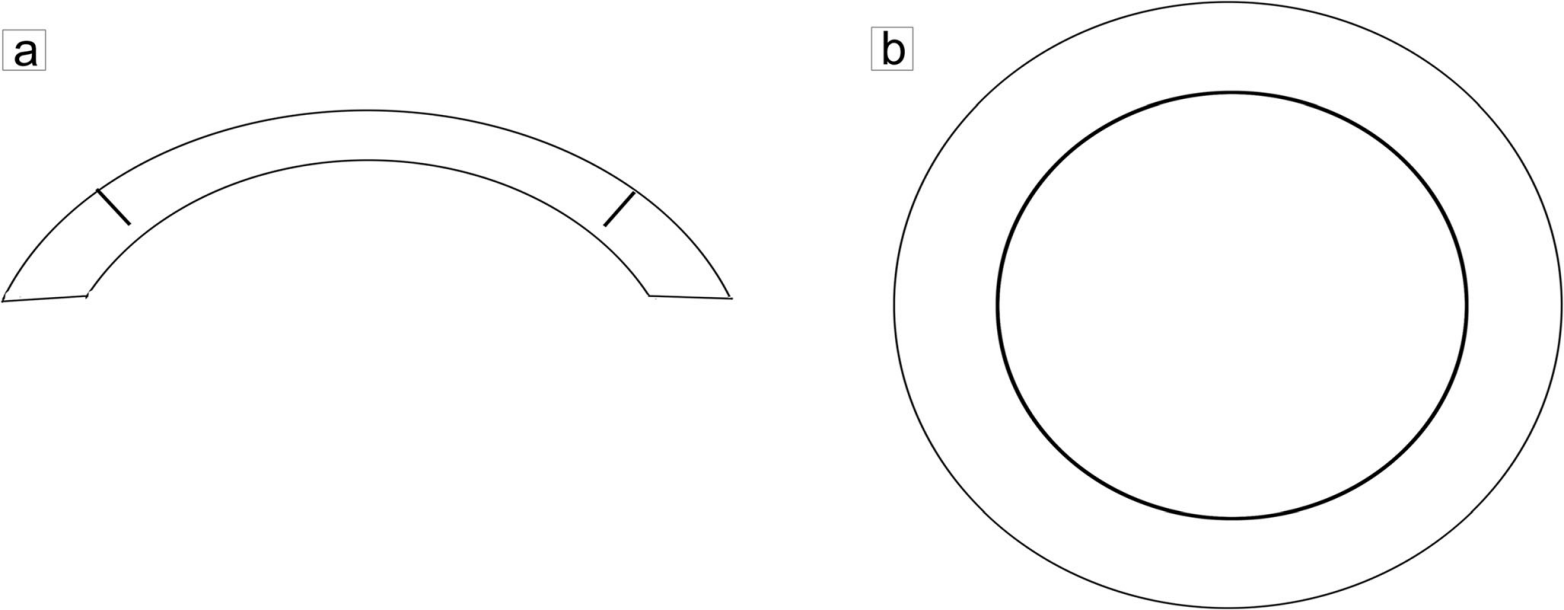
Schematic diagrams of side-cut design. **a** Schematic diagram of longitudinal section of side cut with femtosecond laser. **b** Schematic diagram of transverse section of side cut with femtosecond laser

The FS200 femtosecond laser (Wavelight Laser Technologic AG, Erlangen, Germany) was used for side cutting at 90° with settings of a frequency of 200 kHz and energy of 1.4μJ (Fig. 1a, 1b). The desired graft thickness was set at 110 μm. Each donor cornea was mounted on an artificial anterior chamber prior to measurement of the peripheral corneal thickness. The depth of the side cut was the corneal pachymetry minus 110 μm. The cut diameter, from 8.0 to 8.5mm, was determined according to the size of the recipient’s dissected endothelium.

**Fig. 2:**
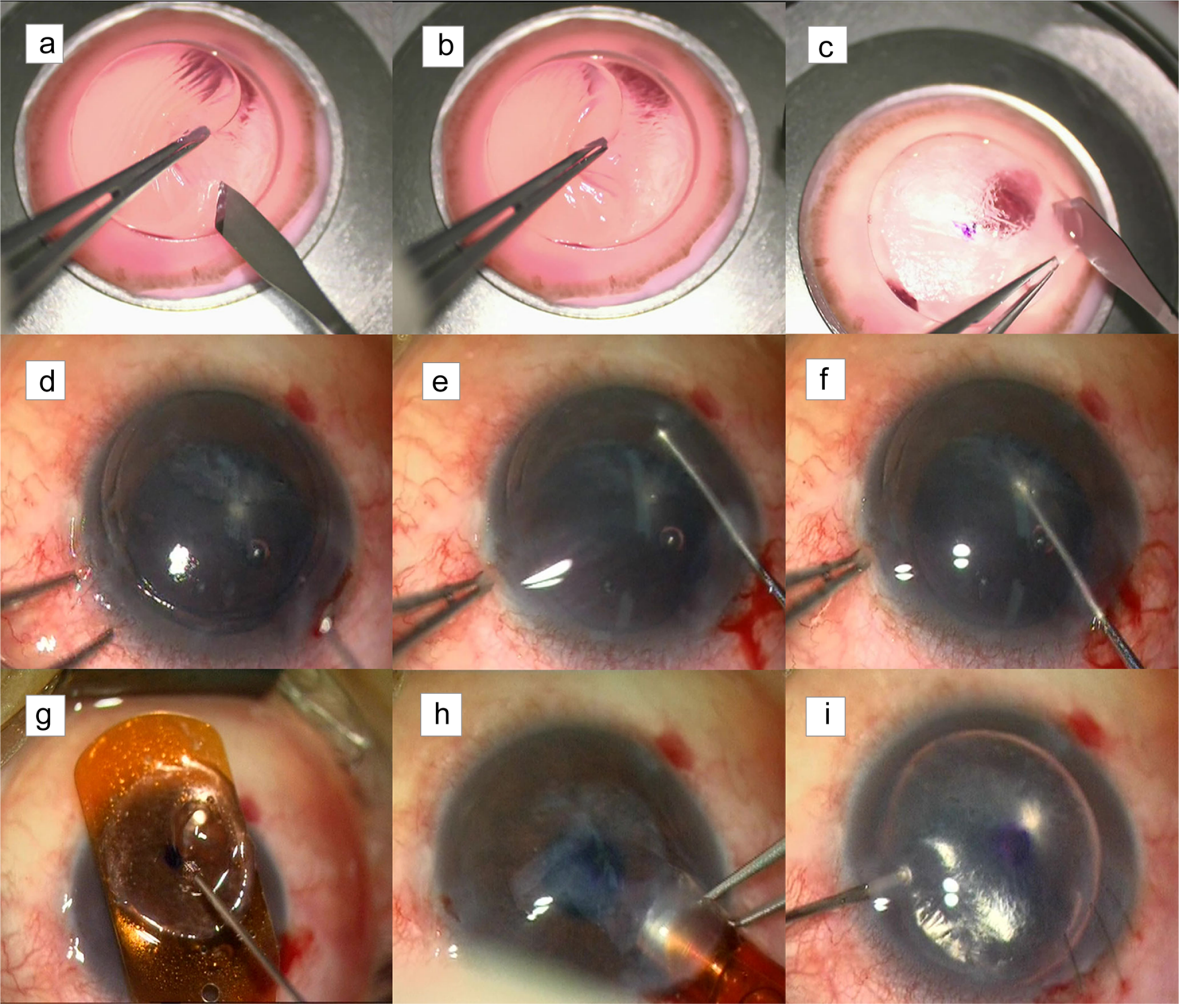
Femtosecond laser semi-assisted Descemet stripping endothelial keratoplasty intraoperative photographs. **a** A manual lamellar cutting of 2-mm corneal periphery; **b** a blunt dissection of the central corneal tissue; **c** cutting off the remained endothelial graft with partial posterior stroma for transplantation; **d** marking on epithelium with a trephine; **e** separating the endothelium along the trephine incision with a Descemet’s membrane stripping hook; **f** removing the endothelium with a hook; **g** putting a graft the endothelial side up on the carrier at the front of the EndoSerter and dropping a small amount of viscoelastic on it. **h** Pushing the folded graft into the anterior chamber through the tunnel incision. **i** Injecting sterile air into the anterior chamber to press the graft

The artificial anterior chamber was filled with Optisol corneal storage medium (Chiron Ophthalmics, Irvine, CA) using a syringe to maintain the chamber pressure within a range from 40 to 50 mmHg for protection of the donor corneal endothelium. After adjustment of the applanation cone and the side cutting area, a corneal incision was created at the corresponding depth using the femtosecond laser. A manual lamellar cutting of 2 mm of the corneal periphery (Fig. 2a) was followed by a blunt dissection of the central 6–7 mm of corneal tissue (Fig. 2b). A marker was created on the stromal side of the endothelial graft. Finally, a 45-degree blade was punctured into the artificial anterior chamber along the side cut track, and the remaining endothelial graft with partial posterior stroma for transplantation was cut off (Fig. 2c).

### Recipient preparation

A 7.75- to 8.25mm-diameter epithelial mark, which was determined according to the size of the recipient’s cornea, was created using a trephine (Fig. 2d) to outline the Descemet membrane stripping area, and the epithelium was scraped. A 1-mm diamond knife was used to create corneal limbal incisions at 3 and 7 o’clock, and a 5-mm corneal tunnel incision from 10 o’clock at the limbus was created to enter the anterior chamber. The anterior chamber was filled with viscoelastics to maintain the shape, and then, the Descemet membrane was removed (Fig. 2e, 2f).

### Donor insertion

The prepared endothelial graft was placed with the endothelial side up on the carrier at the front of the EndoSerter (SightLife Surgical, Winston-Salem, NC) and instilled with a small amount of viscoelastics (Fig. 2g). After pulling the end of the EndoSerter, the endothelial graft was folded into it. Then, the viscoelastics was thoroughly washed out of the anterior chamber. A balanced salt solution was injected into the anterior chamber from the side incision to maintain it before the folded graft was pushed into it through the 5 mm tunnel incision (Fig. 2h), which was then closed using three 10-0 nylon sutures. After the anterior chamber was deepened with liquid filling, sterile air was injected to the eye to press the graft to the host cornea (Fig. 2i). Finally, the endothelial graft was adjusted to achieve good attachment of the Descemet stripping area. The patient was sent back to the ward after lying in the supine position for 30 min.

Postoperatively, endothelial cell loss, central endothelial graft thickness, total central corneal thickness, visual acuity, graft survival status, and complications including graft dislocation, high intraocular pressure, and graft rejection were evaluated.

### Statistical methods

Specular microscopy (Konan Medical Inc., Nishinomiya, Japan) was performed to measure the endothelial cell counts. The rate of postoperative endothelial cell loss, which is expressed as a percentage, was calculated as the preoperative donor endothelial cell density (ECD) minus ECD at 3, 6, 12, and 24 months and then divided by the preoperative ECD. The loss rate was also compared with the results of other related studies [15, 16].

The endothelial graft thickness and the total central corneal thickness were measured by optical coherence tomography (Optovue Inc., Fremont, USA). The *t* test was used to compare the total central corneal thickness before surgery and at 24 months after surgery. The decimal vision was transformed into LogMAR for analysis with the nonparametric test to evaluate differences between the visual acuity before and at 24 months after surgery. The Spearman test was used to analyze the correlation between iris-lens diaphragm abnormalities and graft dislocation.

Corneal endothelial graft failure was defined as failed restoration of the corneal transparency or secondary corneal edema, subepithelial blisters, and endothelial decompensation requiring repeated surgery after the restoration of clarity. The survival rate of the corneal endothelial grafts at 24 months after surgery was calculated according to Kaplan-Meier analysis.

SPSS19.0 (SPSS Inc, Chicago, IL) was used for statistical analyses. Basic descriptive statistics were calculated and reported as the percent for categorical data, as the mean ± standard deviation for normally distributed data, or as the median and range for nonnormally distributed data. *P*<.001 was considered statistically significant.

## Results

### Demographics of the study subjects and surgical procedures

A total of 84 patients (44 males and 40 females; 85 eyes) aged 58±15 years (range, 22 to 85 years) were included in this study. The surgical indications and specific procedures performed are presented in Table 1.

**Table 1:** Indications for femtosecond laser semi-assisted Descemet stripping endothelial keratoplasty (FLS-DSEK)

Indications	Surgical procedures	
	Number of eyes receiving FLS-DSEK (%)	Number of eyes receiving FLS-DSEK, phacoemulsification, and IOL implantation (%)
Fuchs’ dystrophy	1 (1%)	18 (21%)
Aphakic corneal edema	16 (19%)	
Pseudophakic corneal edema	21 (25%)	
Prior glaucoma surgery	5 (6%)	1 (1%)
Failed penetrating keratoplasty	8 (9%)	
Vitrectomy	2 (2%)	
Herpes simplex virus endotheliitis	2 (2%)	1 (1%)
Iris corneal endothelial syndrome	3 (4%)	
Other causes of endothelial dysfunction	4 (5%)	3 (4%)

*IOL*, intraocular lens; *FLS-DSEK*, femtosecond laser semi-assisted Descemet stripping endothelial keratoplasty

### ECD, total corneal thickness, and graft thickness

**Fig. 3:**
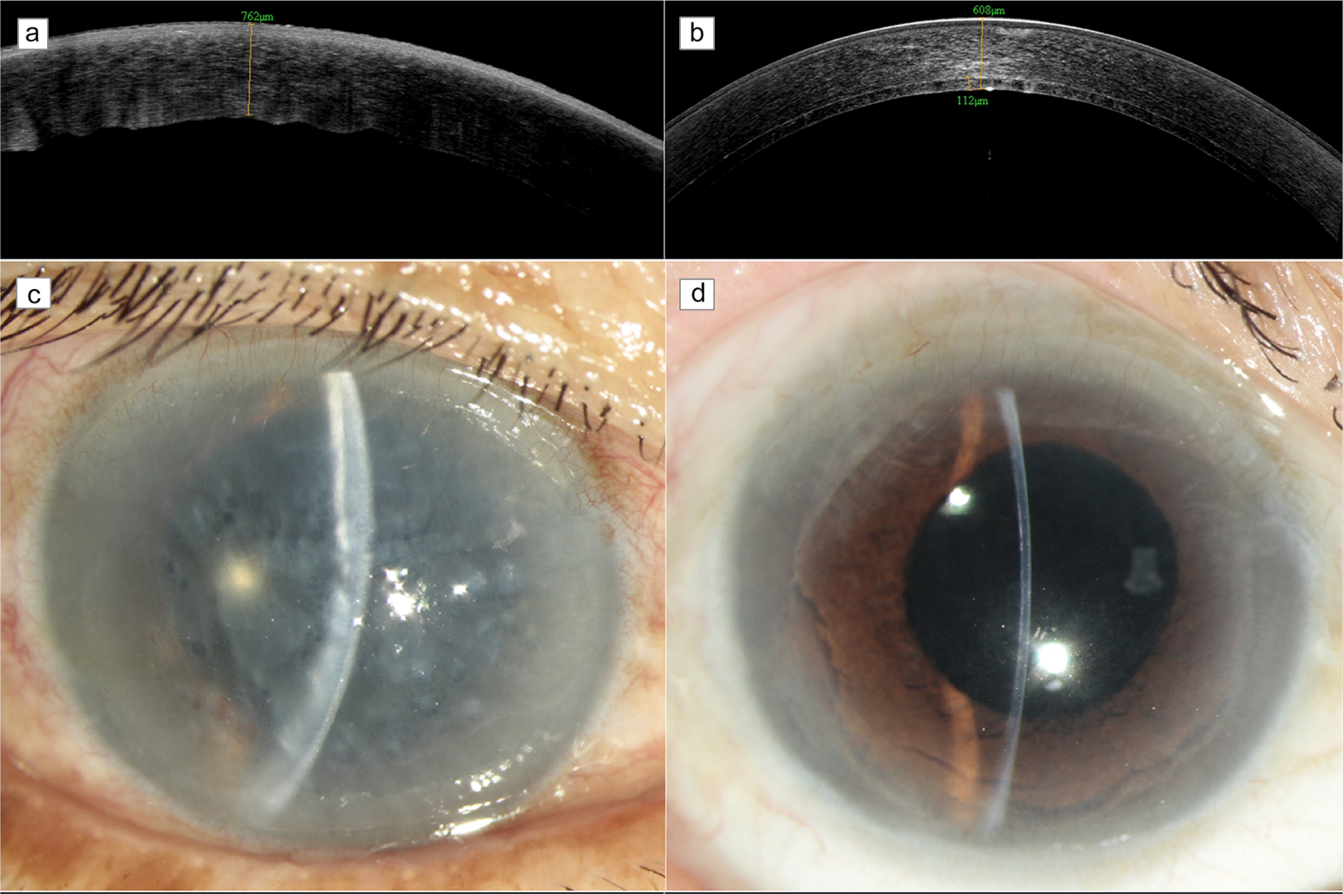
Comparison before surgery and at 24 months after surgery. **a** Optical coherence tomography shows corneal edema and thickening, epithelial edema and subepithelial blisters before surgery. **b** At 24 months after femtosecond laser semi-assisted Descemet stripping endothelial keratoplasty (FLS-DSEK), the grafts are seen well attached to the beds and the corneal edema is significantly alleviated. **c** Corneal edema and subepithelial blisters are observed before surgery. **d** At 24 months after FLS-DSEK, the cornea restores transparency and the graft is in position

The donor corneal ECD was 2411±264 cells/mm^2^ before surgery, and 1990±422 cells/mm^2^, 1958±489 cells/mm^2^, 1932±445 cells/mm^2^, and 1765±387 cells/mm^2^ at 3, 6, 12, and 24 months, with a cell loss rate of 17.4%, 18.8%, 19.9%, and 26.7%, respectively. The endothelial cell loss was most obvious at 3 months after surgery. Due to the role of endothelial cells, corneal edema was gradually alleviated. The total central corneal thickness was 572±91 μm at 24 months, which was significantly thinner than before surgery (802±164 μm) (*t*=4.73, *P*<.001). Moreover, the endothelial graft thickness decreased with time (Fig. 3a, 3b). At 3, 6, 12, and 24 months after surgery, the central corneal thickness of patients was (640 ± 110) μm, (615 ± 85) μm, (608 ± 72) μm, (572 ± 91) μm, and the central graft thickness was (113±54) μm, (102±40) μm, (101±28) μm, and (96±23.2) μm, respectively, as shown in Table 2.

**Table 2:** Changes in endothelial cell counts and graft thickness after femtosecond laser semi-assisted Descemet stripping endothelial keratoplasty (FLS-DSEK)

	Endothelial cell density (cells/mm^2^)	Endothelial cell loss rate (%)	Endothelial graft thickness (μm)	Total corneal thickness (μm)
At 3 months	1990±422	17.4	113±54	640±110
At 6 months	1958±488	18.8	102±40	615±85
At 12 months	1932±445	19.9	101±28	608±72
At 24 months	1765±387	26.7	96±23	572±91

*FLS-DSEK*, femtosecond laser semi-assisted Descemet stripping endothelial keratoplasty

### Visual acuity

All eyes had corneal edema and subepithelial blisters before surgery (Fig. 3c); the cornea transparency was restored after surgery (Fig. 3d). The median of the best-corrected visual acuity (BCVA) in all eyes was 2 logMAR (range, 0.2 to 3 logMAR) before surgery, 74 (87%) of which had values >1 logMAR, and it was significantly improved at 24 months (median, 0.4 logMAR) (*T*=187.5, *P*<.001).

### Complications

None of the 85 eyes suffered intraoperative complications. No perforation occurred during graft preparation, and endothelial grafts were successfully obtained from all 85 donor corneas. Postoperatively, partial graft dislocation was observed in 12 eyes (14%), 10 of which were treated with sterile air injection under topical anesthesia and two with repeated injection, after which the grafts were observed to be well attached to the beds. The rate of partial graft dislocation was 31% (9 in 29 eyes) in the eyes with an abnormal iris-lens diaphragm and 5.4% (3 in 56 eyes) in eyes with a normal iris-lens diaphragm. The partial graft dislocation was closely related to iris-lens diaphragm abnormalities, including aphakia, iris atrophy, and pupillary iris defects (*r*=0.35, *P*<0.001).

Five eyes developed high intraocular pressure, which was controlled with local medical therapy in four eyes and trabeculectomy at 4 months after surgery in one eye whose response to medication was not well. Four eyes showed endothelial graft rejection (on 32 days, 46 days, 44 days, and 90 days after surgery, respectively) with keratic precipitates observed on slit lamp microscopy (Online resource 1a). After local and systemic anti-rejection treatment, the keratic precipitates disappeared **(**Online resource 1b). The endothelial cell damage caused by graft immune rejection was irreversible. In these four eyes, the rate of the mean endothelial cell loss after the occurrence of rejection was 20.6% compared with the cell counts at the final follow-up before rejection. One eye suffered a pupillary block on the first day after surgery, and it was improved following a surgical intervention of anterior chamber reconstruction combined with peripheral iridectomy.

### Graft survival

The endothelial graft failed in the two eyes. The graft survival rate was 98% (*n*= 83) at 24 months (Online resource 2). One eye had previously undergone cataract surgery, but the implanted IOL was dislocated, after which suspended IOL implantation was performed but endothelial dysfunction occurred, and the graft failed at 13 months after FLS-DSEK. Another eye had FLS-DSEK for endothelial dysfunction related to Fuchs’ dystrophy, and graft failure occurred at 11 months. For these two eyes, a second endothelial keratoplasty was performed.

## Discussion

The aim of this study was to investigate the efficacy of the new technique FLS-DSEK. In this study, we studied the existing surgical methods and modified the technique of graft preparation in DSEK by avoiding bottom cutting with femtosecond laser and obtained postoperative endothelial grafts as thin as approximately 100 μm with minimum damage to the endothelium. The graft survival rate was high, while the graft endothelial loss rate was low at 2 years after FLS-DSEK.

In China, due to the difficulty in obtaining donor corneas [7], FS-DSEK is preferred for the treatment of endothelial dysfunction in most eye centers because of the high success rate of graft preparation [8]. The thicker the graft, the less damage the laser causes to the endothelium, so currently, if the entire graft preparation is completed using femtosecond, the graft thickness is expected to be greater than 150 μm [14, 17]. However, it was reported that better postoperative BCVA can be achieved when the endothelial grafts are less than a certain thickness [18, 19]. Ultrathin Descemet stripping automated endothelial keratoplasty (DSAEK), for which the postoperative graft thickness is approximately 100 μm, can even achieve visual acuities comparable to DMEK [10]. However, ultrathin DSAEK has an obvious disadvantage of uneven graft thickness and DMEK shows a high failure rate in graft preparation.

We set the thickness of the grafts according to the following aspects. First, we wanted to improve the success rate of graft preparation as much as possible and to retain a part of the stroma. Considering the failure rate of 2.1% to 29% in cutting an ultrathin endothelial graft (defined as not more than 100 μm) using a microkeratome [10, 20], it is better to use an endothelial graft greater than 100 μm. Second, we wanted to reduce the damage of side cutting to the endothelium. According to Kim et al. [21], fabrication of a corneal ring larger than 100 μm by the femtosecond laser induced the least damage to the endothelial cells at the incision in porcine eyes. Third, the thickness of grafts should be suitable for intraoperative expansion to reduce the intraoperative damage. Terry et al. [18] disclosed that when the graft thickness was greater than 100μm, it was easy to expand the graft in the anterior chamber and avoid injury to the endothelium caused by too many manipulations in the anterior chamber. Fourth, we wanted to minimize the impact of the graft thickness on visual acuity as much as possible. A worse visual acuity has been observed when the thickness of the endothelial graft is greater than 124μm [18]. The thicker the graft, the worse is the visual acuity [22]. Therefore, we decided to set the thickness of the endothelial grafts between 100 and 124μm. In this study, the reserved graft thickness was 110 μm, and manual stripping and blunt dissection were combined.

Although DMEK can obtain the thinnest corneal grafts, the preparation procedure is not easy, with a failure rate of 8% [5, 23]. Using a microkeratome to cut an ultrathin endothelial graft can fail at a rate as high as 29%, and the uneven graft thickness, which is thinner in the middle and thicker at the edges, improves the risk of perforation [10, 11, 18]. In our series, there was no failure in the preparation of the 85 implants, with no waste of the limited number of donor corneas. At 1 and 2 years after surgery, the grafts were 101±28 μm and 96±23 μm, respectively, which were thinner than those in routine FS-DSEK and more similar to those in ultrathin DSAEK [24, 25].

Our FLS-DSEK effectively reduced the damage to corneal endothelial cells, and the loss rate was only 17.4%, 18.8%, 19.9%, and 26.7%, respectively at 3, 6, 12, and 24 months. In a multicenter study about the femtosecond laser-assisted endothelial keratoplasty in the Netherlands [13], the endothelial cell loss rate at 3, 6, and 12 months was respectively 56±16%, 61±16%, and 65±12%. With the advancement of equipment and surgical techniques, Feng et al. [8] demonstrated a lower loss rate of 38.6±19.8%, 44.3±18.9%, and 48.9±18.4% at the three time points in a femtosecond laser-assisted DSAEK study. Moreover, the loss rate was 34%, 36%, and 41% at 6, 12, and 24 months after DSEK [16], and 37%, 40%, and 45% at the three time points after DMEK [15]. The rate of endothelial cell loss was 18.8%, 19.9%, and 26.7%, at 6, 12, and 24 months, respectively in our study (Online resource 3). Price et al. [26] also disclosed a loss rate of 32% at 6 months after DSEK. Khor et al. [27] and Elbaz et al. [28] performed DSAEK with the assistance of the EndoGlide and achieved a loss rate of 14.9% and 41.2%, respectively, at 12 months. According to the American Academy of Ophthalmology, DMEK may lead to a loss rate of 33% (25 to 47%) at 6 months [6]. Murta et al. [29] used both femtosecond laser and microkeratome cutting to achieve a graft thickness of 83.1±23.6 μm and an endothelial cell loss rate of 31.2% at 1 month. Rosa et al. [30] performed femtosecond laser- and microkeratome-assisted DSAEK; the graft was 79.6±14.5 μm in thickness, and the loss of endothelial cells was 31.7% at 3 months. Thinner corneal endothelial grafts could only be obtained at the cost of more endothelial cell damage. In our series, side cutting using the femtosecond laser minimized damage to the endothelium in comparison to using the femtosecond to complete the entire graft preparation. Moreover, manual cutting combined with blunt dissection helped to avoid any mechanical damage to endothelial grafts related to a keratome. In our study, the depth of side cutting could guarantee a 360-degree incision of the anterior stroma, and the depth can be adjusted according to the thickness of the cornea; therefore, the thickness of the implant is accurate and uniform compared with microkeratome-prepared corneal grafts. An approximately 1–2 mm area around the grafts was dissected with a microkeratome when the anterior stroma was removed. Because the side cut depth was sufficiently deep to reach the posterior stroma, usually with a loose tissue structure, the central 6–7 mm optical region could be bluntly torn almost along the same fiber layer, and thus, an endothelial graft with a smooth stromal interface was created. Although the relationship between the graft thickness and postoperative visual acuity remains controversial, better postoperative visual acuity can be obtained when the thickness of the graft is less than 124 μm preoperatively [18] or less than 131 μm postoperatively [19]. The thickness of the endothelial grafts in our cases was 96±23 μm at 24 months and the BCVA reached 0.74±0.48 logMAR, demonstrating a significant improvement after surgery (*P*<.001). Compared with the results in previous reports [10, 25, 31], however, the visual acuity was not sufficiently improved, which may be attributed to the complicated conditions of the eyes included in this study.

In the current study, a femtosecond laser was only used for side cutting to minimize the injury to endothelial cells induced by the laser. With femtosecond laser, individualized graft diameter (ranging from 8.0 to 8.5mm) can be accurately determined according to the diameter of the patient’s cornea.

Corneal endothelial graft dislocation is the most common postoperative complication after corneal endothelial transplantation [32, 33]. We noticed that the abnormality of the lens-iris diaphragm was closely related to the dislocation in our study (*r*=0.35, *P*<.001). The rate of graft dislocation was as high as 50% in the earliest ten cases of DSEK [34], 25% after DSAEK [31], and even higher after DMEK (63%) [35]. With improvements in surgical techniques and the greater experience of surgeons, the rate of graft dislocation after DSAEK has decreased to 2% [36]. In our series, the rate was not satisfactory (14.1%), despite the lying in the supine position for 30 min before the patient was sent back to the ward. The statistical analysis showed that this result was correlated with the complicated eye conditions of our patients, with an abnormal lens-iris diaphragm as a high-risk factor (*P*<.001).

From this observational study, we noticed that FLS-DSEK can achieve favorable therapeutic results, but the femtosecond laser parameter settings and operation of upper corneal tissue cutting combined with blunt dissection, thus creating a smooth anterior graft surface, is much more technically challenging than using a microkeratome and may affect the visual acuity, which present high requirements for surgeons. In addition, the procedure is much more costly compared to the methods using a keratome, or DMEK. What is more, in this preliminary study observing this new modified technique itself, the outcomes are satisfying, but a longer follow-up, a large sample analysis, and comparative studies with other surgical methods are needed for a comprehensive evaluation.

## Supplementary Information

Online resource 1 Comparison before and after anti-rejection treatment. **a** Slit lamp microscopy observes endothelial graft rejection with keratic precipitates. **b** After local and systemic anti-rejection treatment, the keratic precipitates disappears and the cornea restores transparency (PNG 3294 kb)

High resolution image (TIF 12178 kb)

Online resource 2 Functions image of endothelial graft survival at 24 months after femtosecond laser semi-assisted Descemet stripping endothelial keratoplasty (FLS-DSEK) treatment for corneal endothelial dysfunction (PNG 5630 kb)

High resolution image (TIF 2724 kb)

Online resource 3 Comparison of endothelial cell survival rate after femtosecond laser semi-assisted Descemet stripping endothelial keratoplasty (FLS-DSEK) with that after conventional Descemet stripping endothelial keratoplasty (DSEK) and Descemet membrane endothelial keratoplasty (DMEK) (PNG 5635 kb)

High resolution image (TIF 3392 kb)

Online Resource 4 Surgery video of femtosecond laser semi-assisted Descemet stripping endothelial keratoplasty: endothelial graft preparation and donor insertion (MOV 15952 kb)

## Data Availability

The datasets generated during and/or analyzed during the current study are available from the corresponding author on reasonable request.
