# Supplementary figures and images for: Femtosecond laser semi-assisted Descemet stripping endothelial keratoplasty: 2-year outcomes of endothelial cell loss and graft survival
Source: Graefes Arch Clin Exp Ophthalmol. 2021 Aug 31;260(1):181–9. doi: 10.1007/s00417-021-05383-x (PMC8763781; doi:10.1007/s00417-021-05383-x)

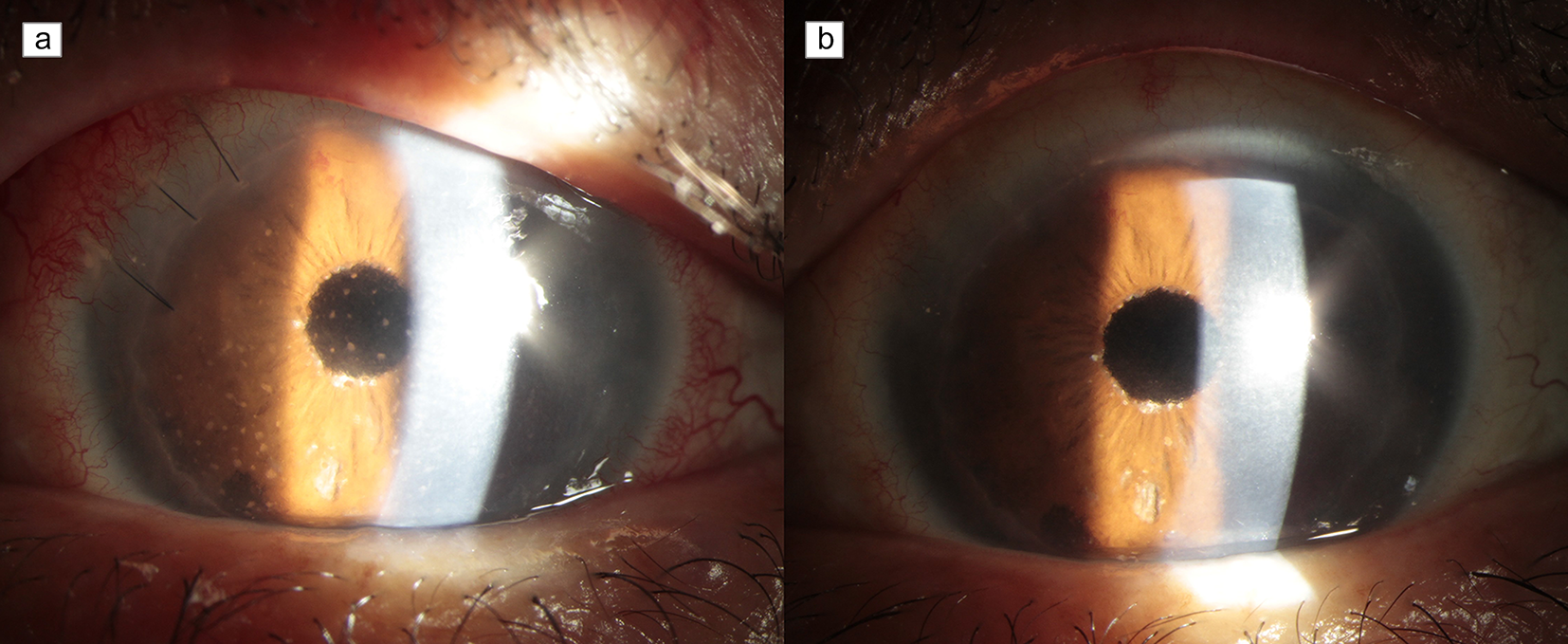

Supplement: Supplementary file 1 — Comparison before and after anti-rejection treatment. a Slit lamp microscopy observes endothelial graft rejection with keratic precipitates. b After local and systemic anti-rejection treatment, the keratic precipitates disappears and the cornea restores transparency (PNG 3294 kb) [file 417_2021_5383_Fig4_ESM.png]

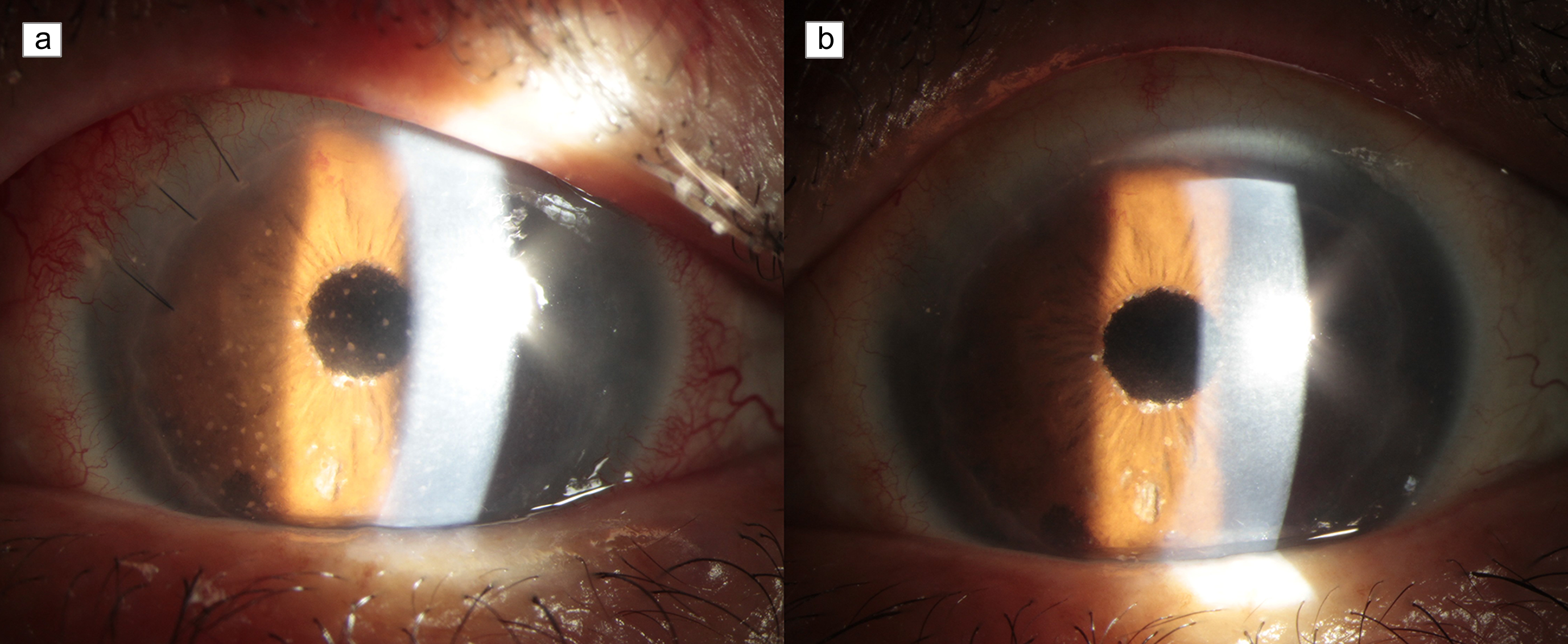

Supplement: Supplementary file 2 — High resolution image (TIF 12178 kb) [file 417_2021_5383_MOESM1_ESM.tif]

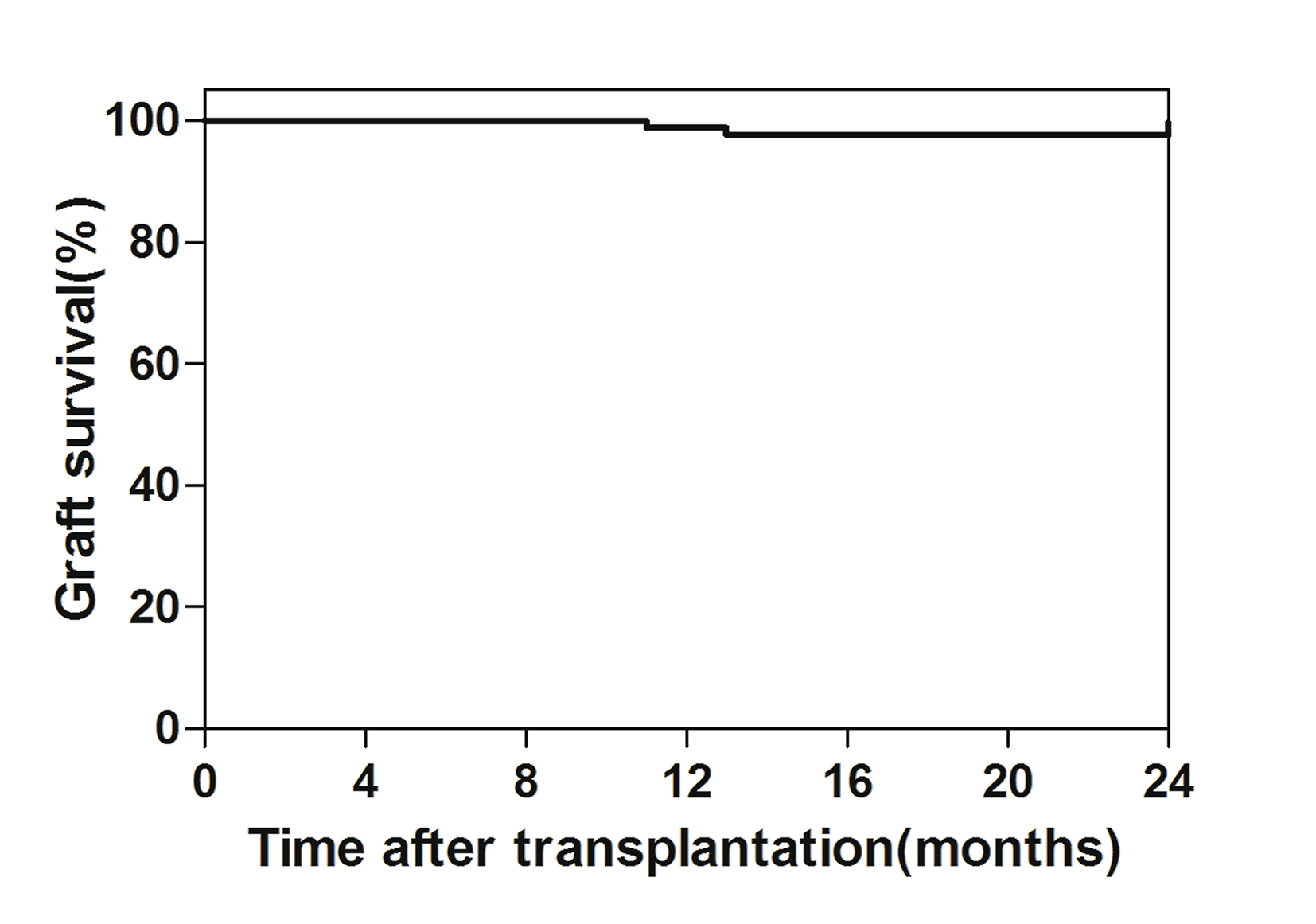

Supplement: Supplementary file 3 — Functions image of endothelial graft survival at 24 months after femtosecond laser semi-assisted Descemet stripping endothelial keratoplasty (FLS-DSEK) treatment for corneal endothelial dysfunction (PNG 5630 kb) [file 417_2021_5383_Fig5_ESM.png]

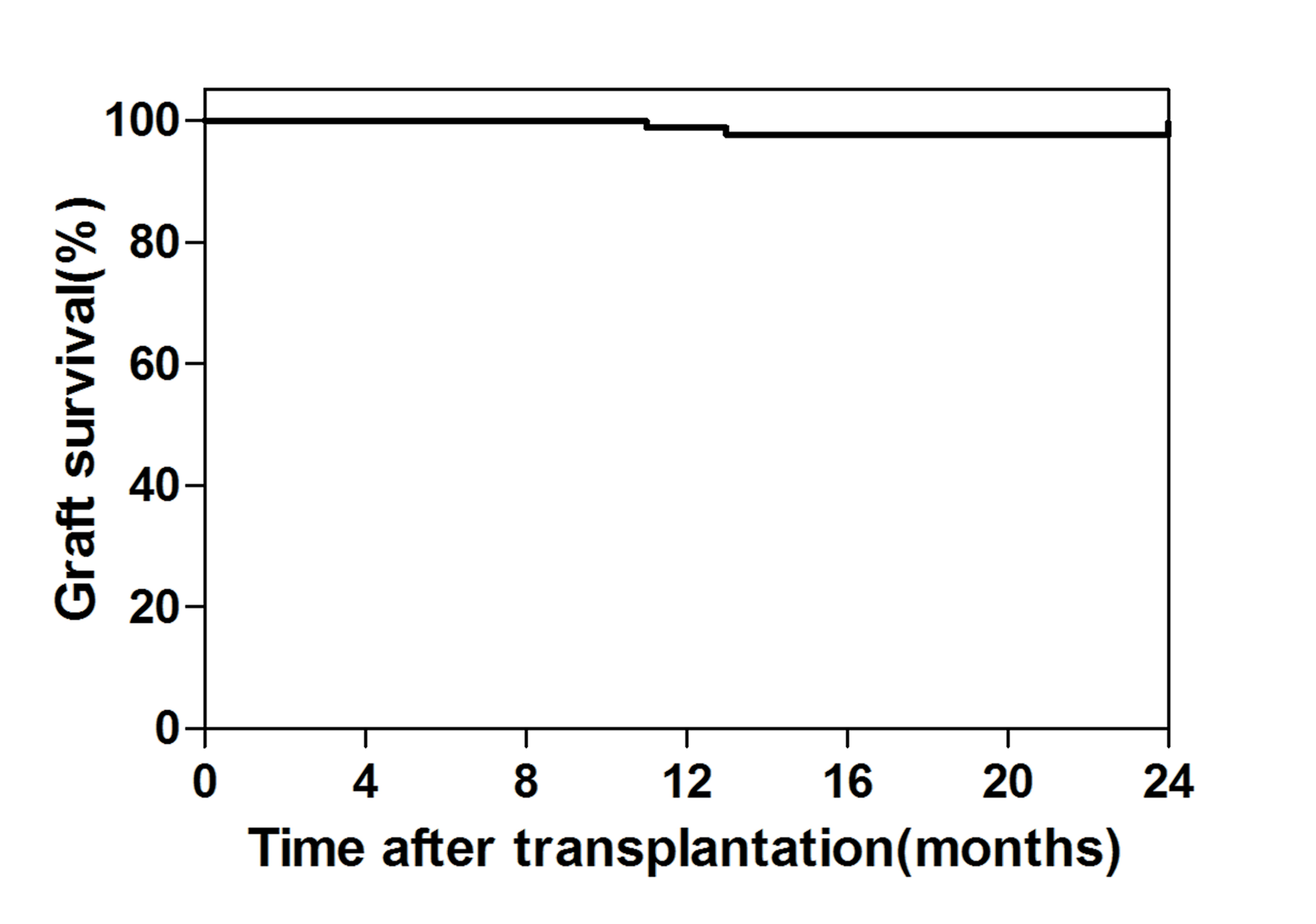

Supplement: Supplementary file 4 — High resolution image (TIF 2724 kb) [file 417_2021_5383_MOESM2_ESM.tif]

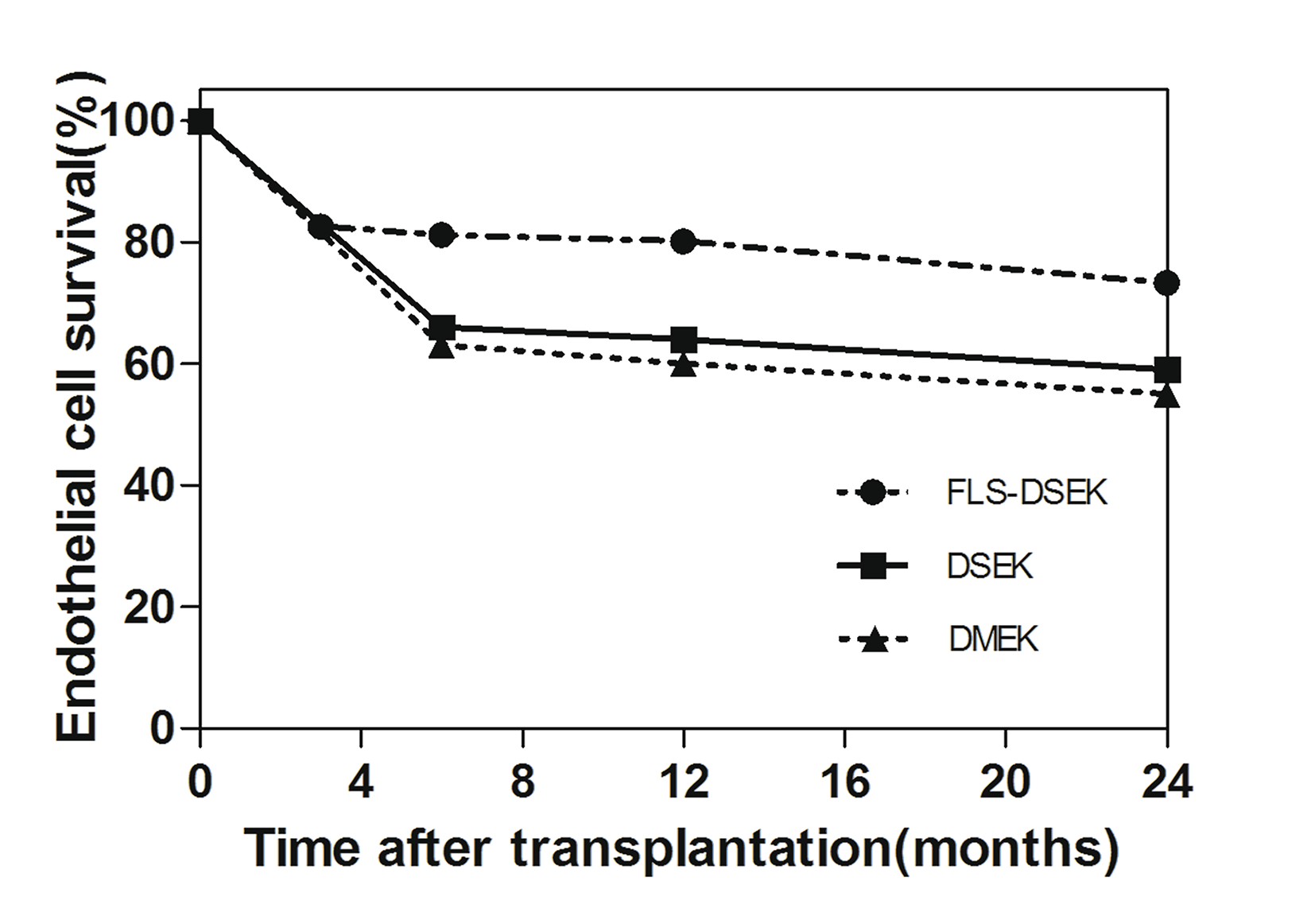

Supplement: Supplementary file 5 — Comparison of endothelial cell survival rate after femtosecond laser semi-assisted Descemet stripping endothelial keratoplasty (FLS-DSEK) with that after conventional Descemet stripping endothelial keratoplasty (DSEK) and Descemet membrane endothelial keratoplasty (DMEK) (PNG 5635 kb) [file 417_2021_5383_Fig6_ESM.png]

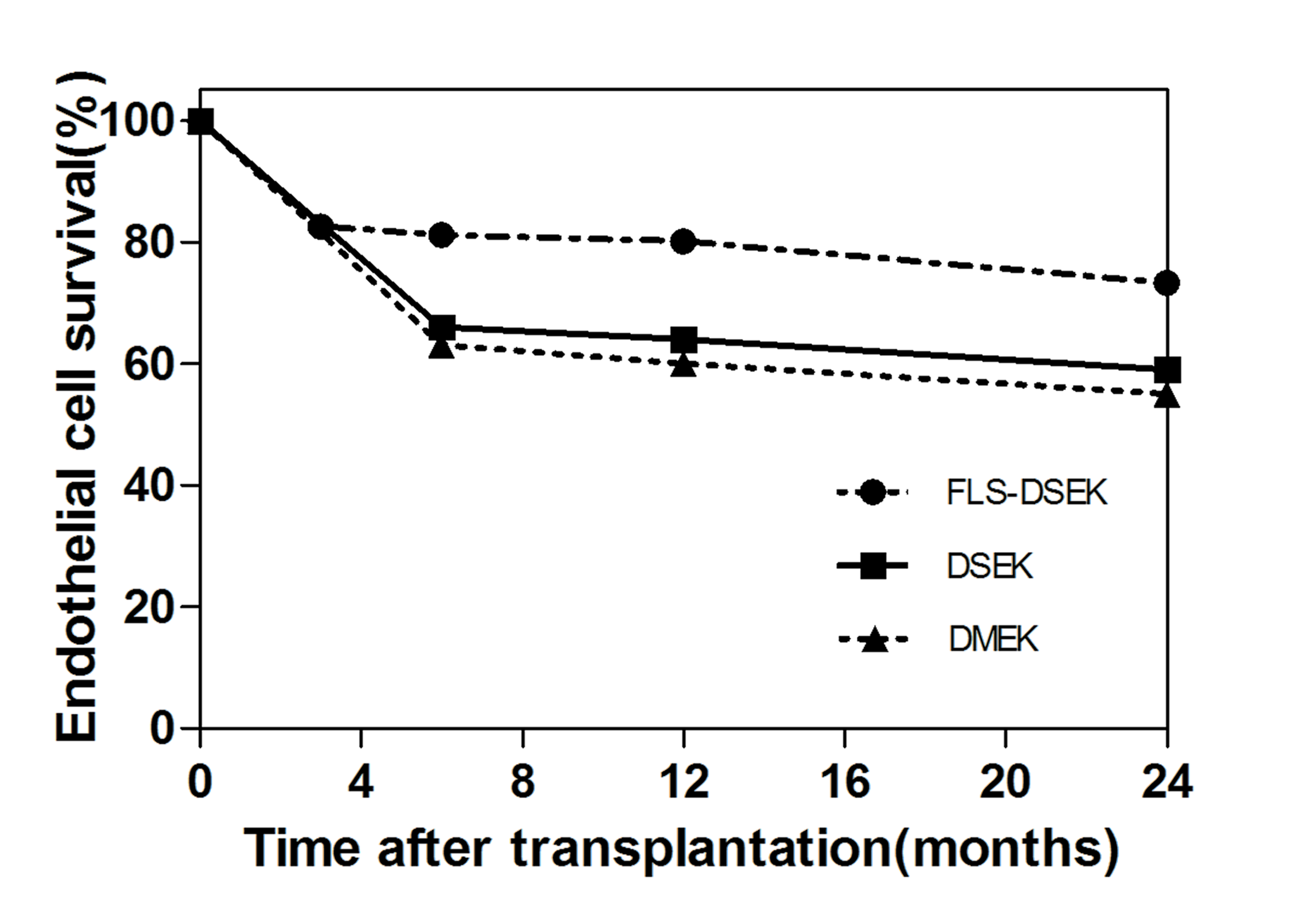

Supplement: Supplementary file 6 — High resolution image (TIF 3392 kb) [file 417_2021_5383_MOESM3_ESM.tif]
